# Autologous Platelet Concentrates and Photobiomodulation as Biologically Active Modifiers of Hard and Soft Tissue Healing: A Randomised Controlled Trial

**DOI:** 10.3390/jfb17030127

**Published:** 2026-03-05

**Authors:** Daniel Selahi, Marzena Dominiak, Wojciech Niemczyk, Artur Pitułaj, Kamil Jurczyszyn, Jakub Hadzik

**Affiliations:** Department of Dental Surgery, Faculty of Dentistry, Wroclaw Medical University, Krakowska 26, 50-425 Wroclaw, Poland

**Keywords:** third molar extraction, photobiomodulation, platelet-rich fibrin, bone regeneration, wound healing, randomised controlled trial

## Abstract

Background/Objectives: This study evaluated autologous platelet concentrates (APCs), including advanced platelet-rich fibrin (A-PRF+) and concentrated growth factors (CGFs), as biologically active matrices, and photobiomodulation (PBM) as a biophysical stimulus affecting soft and hard tissue regeneration following mandibular third molar extraction. Methods: A six-arm parallel randomised controlled trial was conducted including 135 patients. A total of 122 participants completed follow-up and were analysed: control (*n* = 22), photobiomodulation (*n* = 20), A-PRF+ (*n* = 19), CGF (*n* = 20), A-PRF+ plus photobiomodulation (*n* = 22), and CGF plus photobiomodulation (*n* = 19). The primary endpoint was postoperative pain intensity assessed on postoperative day 3 using an 11-point visual analogue scale (VAS). Secondary outcomes included swelling, trismus, wound healing assessed by the early healing index, and bone regeneration assessed by CBCT-based fractal dimension analysis at 4 months. Results: On postoperative day 3, mean VAS pain was 2.95 ± 2.65 in the control group and 1.00 ± 1.65 in the photobiomodulation group, corresponding to a mean difference of 1.95 VAS points. The overall between-group difference for day 3 pain was statistically significant. In swelling outcomes, no statistically significant between-group differences were observed at days 1, 3, or 7 across facial measurement lines. In CBCT fractal analysis, a significant group effect was detected for the mid socket region, with higher fractal dimension at 4 months in the CGF plus photobiomodulation group compared with the control. Conclusions: Both APCs and PBM positively influenced postoperative healing. Their combined application, particularly CGF with PBM, showed the most consistent regenerative effects, although not all outcomes differed significantly between groups. These minimally invasive strategies may support soft and hard tissue regeneration.

## 1. Introduction

### 1.1. Rationale

Impacted third molar surgery is a common procedure in the field of dentistry. The third molar is characterised by a higher rate of developmental abnormalities, an unsuitability for soft tissue environments, and a lack of access to oral hygiene. Indications for the extraction of third molars may include the presence of dental caries, periodontal disease, orthodontic treatments, pericoronitis, cysts, and tumour formation associated with the impacted teeth [1,2]. Although impacted tooth extraction is the most frequently performed surgical procedure in the field of maxillofacial surgery, the incidence of complications is also one of the highest. It should be noted that not all impacted teeth have the potential to cause problems [3]. The period of convalescence following the surgical extraction of an impacted third mandibular molar is characterised by an intense inflammatory response. This process is responsible for the occurrence of postoperative pain, facial swelling, and trismus, which have a detrimental effect on the patient’s quality of life for a period of 7–10 days after the surgery [4]. Numerous factors have the capacity to exert an adverse effect on the healing period of patients [5,6,7]. However, a plethora of therapeutic interventions have been developed with the objective of promoting healing and counteracting these negative factors [8,9,10,11,12,13]. The conventional therapeutic approach to mitigate postoperative complications involves the administration of medications such as nonsteroidal anti-inflammatory drugs (NSAIDs), corticosteroids, and analgesics. However, despite their efficacy, these pharmaceutical agents can induce significant adverse effects, including the propensity for systemic bleeding, gastrointestinal irritation, and allergic reactions. Antibiotics have been shown to reduce the risk of postoperative infection and alveolitis; however, their administration is only recommended in selected cases because of the possibility of developing bacterial resistance [14,15]. The necessity for alternative and innovative methods for the resolution of the symptomatology following third molar extraction is evident from these considerations, and such methods should ideally have no adverse effects [14]. Among such methods are photobiomodulation (PBM; low-level laser therapy, LLLT) and autologous platelet concentrate (APC) therapy [16,17].

Low-level laser therapy (LLLT) is a technique involving the use of low-power, non-ionised red and infrared light sources to reduce inflammation, accelerate healing and reduce pain and discomfort in a variety of clinical conditions [18]. It has been demonstrated that red and infrared lasers, with wavelengths of approximately 660 and 808 nm, respectively, have the capacity to stimulate the synthesis of growth factors and the process of angiogenesis. This, in turn, can facilitate the healing of wounds [19,20]. Platelet-rich fibrin (PRF) is a second-generation platelet concentrate that was first described by Choukroun et al. in 2000 [21]. In the process of wound healing, platelets have been shown to play a pivotal role. Once activated, they secrete a range of factors that stimulate cell proliferation, including platelet-derived growth factor (PDGF), transforming growth factor (TGF-β), and insulin-like growth factor I (IGF-I). Furthermore, they secrete fibrin, fibronectin, and vitronectin, which form a matrix for connective tissue and facilitate the migration of cells by acting as adhesion molecules. Consequently, platelets exert a pivotal influence on processes such as cell proliferation, collagen synthesis, and osteoid formation [22,23]. Concentrated growth factors (CGFs), a product developed by Sacco in 2006, represent the latest generation of platelet concentrate products [20]. The repeated adjustment of the centrifugation speed results in the availability of CGFs as a modified form of PRF, characterised by high concentrations of cytokines, platelets, nucleated cells, and a dense fibrin scaffold [24].

### 1.2. Objectives

The objective of this study was to evaluate the effectiveness of autologous platelet concentrates (CGF or PRF) and laser photobiomodulation in enhancing bone and soft tissue healing following mandibular third molar extraction. A randomised controlled trial was conducted to assess clinical and radiological outcomes, including pain levels, swelling, trismus, wound closure, and fractal analysis of bone regeneration. The study sought to determine whether these regenerative techniques offer significant advantages over conventional healing processes. A predefined hierarchy of endpoints was established prior to statistical analysis to reduce the risk of type I error related to multiple comparisons.

### 1.3. Hypothesis

The study’s authors posited the following hypotheses:

1. The least pain in patients after surgery occurs after the use of CGFs and photobiomodulation.
2. The greatest postoperative swelling is found in patients who do not use tissue engineering methods or photobiomodulation.
3. The smallest difference in preoperative and postoperative jaw dilation width is found in patients treated with CGFs and photobiomodulation.
4. The values of the fractal dimensions of the alveolar filling bone 4 months after extraction relative to the reference bone are the most similar to each other when CGFs and photobiomodulation are used.

## 2. Materials and Methods

### 2.1. Trial Design

The clinical trial was a prospective, single-centre, six-arm, parallel-group randomised controlled trial. Approval for the study was obtained from the Bioethics Committee at the Wroclaw University of Medical Sciences (protocol number KB7052019-UMW; approval ID KB-705/2019) on 22 October 2019. The study protocol was planned in accordance with the Consolidated Standards of Reporting Trials (CONSORT) guidelines.

The trial was registered at ClinicalTrials.gov (Identifier: NCT07324213) under the title “Healing of Mandibular Third Molar Extraction Sockets Using Platelet Concentrates and Photobiomodulation”. The study was conducted at the Medical Innovation Center Wrocław.

The trial was carried out in accordance with the principles of the Declaration of Helsinki and Good Clinical Practice (GCP) guidelines. All participants provided written informed consent prior to enrolment. The study protocol and outcome measures were predefined before patient recruitment and remained unchanged throughout the study period [25].

### 2.2. Participants

A total of 135 healthy young adults were enrolled in the study, with no gender-based restrictions imposed on eligibility. All patients meeting the inclusion criteria were invited to participate. This was determined through a combination of clinical and radiological examinations, including orthopantomographic (OPG) radiographs and cone beam computed tomography (CBCT) scans as necessary for comprehensive diagnostic imaging. Exceptionally difficult impactions were excluded to minimise variability in surgical trauma and operating time, which can independently affect postoperative pain, swelling, and tissue healing. The inclusion and exclusion criteria are presented in Table 1.

#### Sample Size Rationale

The sample size rationale was based on the primary endpoint, defined as postoperative pain intensity (VAS, 0 to 10) assessed on postoperative day 3. A clinically meaningful between-group difference for postoperative pain was assumed to be 2.0 points on the VAS at day 3. Based on published postoperative pain variability after mandibular third molar extraction and local clinical experience, the standard deviation was assumed to be approximately 2.3 points. With a two-sided alpha level of 0.05 and 80% power, the required sample size for a key pairwise comparison (control versus an active intervention group) was approximately 20 participants per group.

Given the six-arm design, the planned minimum enrollment was therefore 120 participants (20 per group). To account for an anticipated loss to follow-up of approximately 10%, the recruitment target was set at 132 participants (22 per group). Ultimately, 135 participants were enrolled and randomised, and 122 participants completed all follow-up visits and were included in the final analysis. The analysed sample corresponded to approximately 18 to 22 participants per group, which was close to the intended target but may have limited the ability to detect small between-group differences, particularly across multiple secondary outcomes and time points.

### 2.3. Study Groups

**Figure 1:**
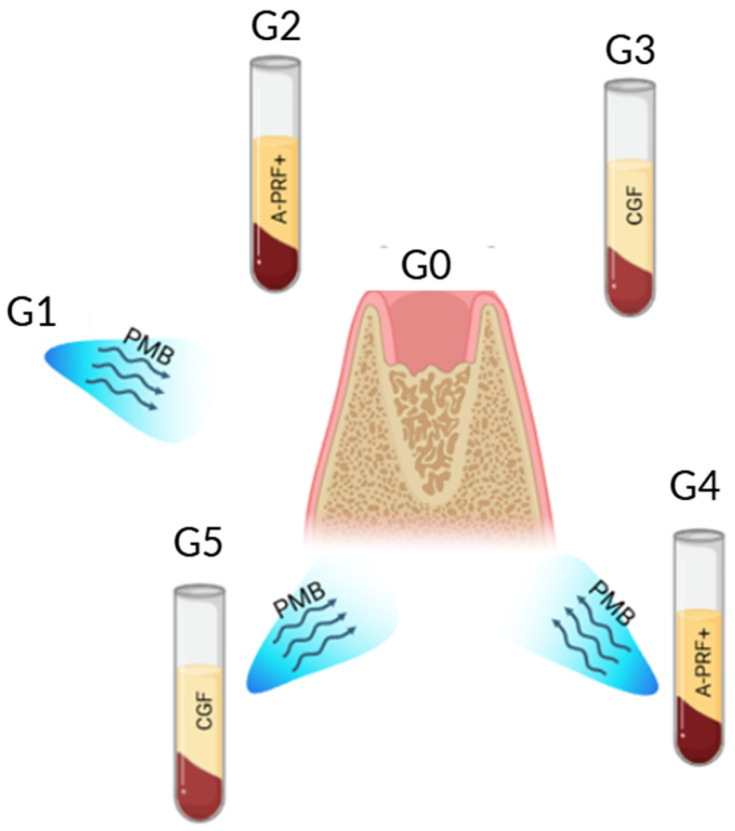
Graphical representation of the groups studied in the trial.

Patients who met the inclusion criteria were randomly assigned to one of six groups, which were created for this study (Figure 1):

- G0 (control group): Extraction of the mandibular third molar without the use of APC and photobiomodulation.
- G1: Extraction of mandibular third molar with photobiomodulation.
- G2: Extraction of mandibular third molar with the application of A-PRF+.
- G3: Extraction of mandibular third molar with the application of CGF.
- G4: Extraction of mandibular third molar with the application of A-PRF+ and use of photobiomodulation.
- G5: Extraction of mandibular third molars with the application of CGF and use of photobiomodulation.

### 2.4. Autologous Platelet Concentrates Preparation

Sterile vacuum glass tubes (CDRICH) with a capacity of 10 mL and dimensions of 16 × 100 mm were used for both PRF and CGF preparation. The tubes did not contain any additives. In each patient in groups G2, G3, G4, and G5, 20 mL of venous blood was collected. Immediately after blood collection, the tubes were placed in centrifuges. A PRF Duo System^®^ centrifuge (PROCESS FOR PRF, 49 Rue Gioffredo, 06000 Nice, France) with the centrifugation cycle set at 1300 rpm [~200 g RCF max (~130 g RCF clot)] was used to obtain A-PRF+, the duration to obtain A-PRF+ was 8 min. A Medifuge CGF MF 200^®^ centrifuge was used (Silfradents.r.l., Via G. di Vittorio 35/37, 47018 S. Sofia (FC), Italy), with 4 centrifugation parameters during one cycle lasting 14 min. The course of the 4 cycles of proctal CGF manufacture was as follows: 30′ acceleration, 2′ 2700 rpm, 4′ 2400 rpm, 4′ 2700 rpm, 3′ 3000 rpm and 36′ deceleration until end. Following the process of centrifugation, the test tubes were removed from the centrifuge. A minimum of five minutes was allowed to elapse before the fibrin clot was removed, in order to ensure that it underwent adequate cross-linking. When removing the clots from the tubes, they were cut off approximately 2 mm below the erythrocyte zone to ensure that the zone most rich in growth factors was not wasted.

### 2.5. Interventions

The procedure was initiated with the administration of block and infiltration anaesthesia in the oral vestibule of the area designated for extraction, employing Weissbrem’s method of anaesthesia. This was composed of 4% articaine with adrenaline in a ratio of 1:100,000 (Septanest^®^, Septodont, Saint-Maur-des-Fossés, France), with a total of two ampoules being utilised prior to the commencement of the procedure. The potential for supplemental anaesthesia for the patient during the extraction process was considered. A full-thickness flap was then detached buccally from the tooth to be extracted with a release cut on the mandibular branch, guided through the pocket up to the mesial surface of the second molar. If necessary, the cut was extended to the mesial surface of the first molar. The tooth was then extracted using straight levers with or without rotary instruments on the contra-angle for coronal-root separation or on the handpiece to abolish the alveolar bone (only in the case of deeper retained teeth). The surgical site was then washed profusely with saline solution and the remains of the developmental bellows or, if a bone pocket is present, the inflammatory granulation filling it were removed. Post-extraction, the alveoli were either filled with blood seeping from the bone and soft tissues or with an APC of choice and sutured blind to reduce contact of the damaged tissues with the oral cavity lumen. The sutures were removed after seven days. We conducted analgesic standardisation and recording. Postoperative analgesia was standardised by prescribing nimesulide (Aulin^®^, Angelini Pharma, Ancona, Italy) 100 mg every 12 h for 3 days for all participants unless contraindicated. Participants were instructed to record each administered dose in a standardised medication diary. In addition, a predefined rescue analgesic (paracetamol 500 mg, up to 3 g per day) was permitted if pain was not adequately controlled; rescue intake (dose and timing) was recorded in the same diary and reviewed at each follow-up visit. Any deviations from the planned NSAID schedule and any additional analgesics were documented. Patients in groups G1, G4 and G5 were treated with the photobiomodulation regimen described below. Postoperative management included standard oral hygiene instructions aimed at minimising bacterial load in the surgical area, including gentle rinsing, avoidance of mechanical irritation of the wound, and adherence to routine postoperative care recommendations. These measures were intended to limit microbial interference with soft tissue healing and bone regeneration, particularly in the early postoperative period.

Due to the fact that the exclusion criteria were systemic diseases and very difficult cases of impacted teeth, in accordance with the current guidelines on antibiotic therapy [26], none of the patients were prescribed antibiotics after the procedure.

#### Photobiomodulation Protocol

Photobiomodulation was performed using a Lasotronix Smart M^®^ diode laser (Lasotronix Sp. z o.o., Piaseczno, Poland) emitting red light at a wavelength of 635 nm. The device operated in continuous wave mode with a constant output power of 100 mW. The irradiation was delivered intraorally directly into the extraction socket using a fibre optic applicator. The beam was applied using a non-contact technique, held perpendicular to the tissue surface at an approximate distance of 1 to 2 mm without compression of soft tissues. Each session consisted of four irradiation points within the extraction socket: buccal, lingual, distal, and central. Each point was irradiated for 20 s. At an output power of 100 mW, the energy delivered per point was 2 J. The target energy density was 4 J/cm^2^ per point. The total energy delivered per session per extraction socket was therefore 8 J. Photobiomodulation sessions were performed according to a predefined schedule:

1. Immediately after suturing on the day of surgery.
2. Postoperative day 3.
3. Postoperative day 7 prior to suture removal.

This three-session protocol was applied consistently to all patients in groups receiving PBM. All procedures were performed by the same trained operator to ensure standardisation. Both the patient and operator wore protective eyewear during irradiation.

### 2.6. Outcome Assessment

#### 2.6.1. Clinical Parameters

For the purpose of analysis, an index of oedema was created for each of the measured lines by subtracting from the length of the line examined before treatment the length of the line of the given day after treatment. The overall swelling index used in this study was an internally derived composite measure based on previously validated linear facial measurements and was intended to provide a global and clinically practical assessment of postoperative facial oedema. This was done separately for day 1 after treatment, day 3 after treatment, day 7 after treatment and month 4 after treatment. Three standardised lines were measured using predefined skin landmarks: line A from the outer canthus of the eye to Gonion (Go), line B from the tragus (ear tragus) to Cheilon (Ch, oral commissure), and line C from the tragus to Pogonion (Pg, most anterior point on the chin) [27]. To determine a more general direction of change in swelling, a new index was created: the amount of overall swelling for each of the study periods after mandibular third molar extraction by calculating the average of the line lengths of A, B and C on a given day. The extent of jaw dilation was used to create the parameter of maxillary swelling, defined as the change in the extent of jaw dilation for each of the study periods after extraction by subtracting from the extent of jaw dilation before surgery, the extent of jaw dilation from a given day after extraction: 1st day after surgery, 3rd day after surgery, 7th day after surgery and 4th month after surgery.

After the procedure, patients attended four follow-up appointments. Follow-up visits were scheduled at 1, 3, and 7 days and at 4 months after extraction. In addition, on the day of the procedure, the clinically assessed measures were

- Duration of treatment (in minutes);
- Extraction of the tooth in its entirety or using resection methods;
- Type of postoperative wound closure—complete or incomplete;
- Measurement of wound length (in mm);
- Pain during the procedure assessed by the patient’s subjective feelings measured on an 11-point VAS pain scale, where 0 means no pain and 10 means unbearable pain; pain was assessed after extraction and at all follow-up visits.

At follow-up visits, in addition to the VAS pain score (ranging from 0—no pain to 10—unbearable pain), the following were assessed: jaw opening (in mm), tissue swelling, measurement of wound length (in mm), and the degree of wound closure assessed on the 5-point EHI (early healing index) [28] scale 7 days after surgery, where

1°—Complete wound closure;

2°—Complete wound closure with a fine fibrous line;

3°—Complete closure of the flap but with marked fibrosis;

4°—No complete closure of flap, marked fibrosis;

5°—Open wound, visible soft tissue necrosis.

The incidence of oedema was determined in millimetres by 3 straight lines passing through skin reference points on the face:

- Line A connecting the outer corner of the eye–Gonion, Go (the point located most backwards and downwards on the angle of the jaw).
- Line B connecting the ear slant–Cheilon, Ch (the point marking the corner of the mouth).
- Line C joining the ear crook–Pogonion, Pg (point located most anteriorly on the curve of the chin).

**Figure 2:**
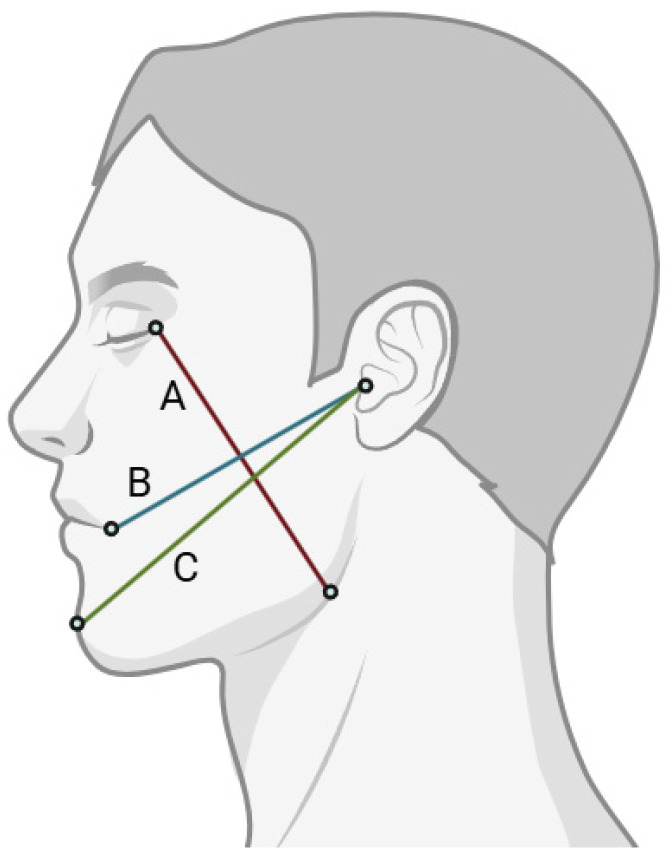
Points and reference lines used to assess oedema.

The above-described segments between reference points are shown in Figure 2.

Postoperative trismus was assessed as maximal mouth opening (maximal interincisal distance), measured in millimetres between the maxillary and mandibular central incisor edges at maximal opening. Measurements were performed with a metal ruler with millimetre grading at baseline and at each follow-up visit, and changes from the baseline were used for analysis.

#### 2.6.2. Radiological Parameters and CBCT Fractal Analysis

Immediately following the procedure and at the final follow-up visit (4 months after extraction), a CBCT scan (Rayscan Alpha device 160^®^, Ray Co, Hwaseong, Korea) was performed. All scans were acquired using a standardised protocol. The following acquisition parameters should be reported exactly as recorded in the CBCT console protocol or DICOM header for this study cohort: voxel size 0.20 mm, tube voltage 90 kVp, tube current 10 mA, and field of view 10 × 10 cm. CBCT datasets were reviewed in RadiAnt DICOM Viewer, version 2022.1.1 (64-bit) (Medixant, Poznań, Poland). For each scan time point, three standardised axial cross sections through the extraction socket were selected to represent the socket apex, mid-alveolar region, and socket entrance. These levels corresponded to the “apex of the alveolus”, “mid-alveolar region”, and “entrance to the alveolus” reported in the outcome tables. ROI preparation was performed by generating screenshots from each selected cross section and uniformly enlarging them in Adobe Photoshop (version 24.6.0). From each cross section, square ROIs of 30 × 30 pixels were cropped to cover the full socket area. The number of ROIs depended on the socket level, ranging from 1 to 2 ROIs at the socket apex up to approximately 20 to 23 ROIs at the socket entrance, to ensure that the full cross-sectional socket area was analysed rather than a single arbitrarily selected fragment.

Fractal analysis was performed in ImageJ version 1.53e using the FracLac plugin version 2.5. The fractal dimension was computed using the box counting method as implemented in FracLac. All preprocessing and FracLac settings were held constant across participants, groups, and time points. The primary fractal outcomes were defined a priori as fractal dimensions at three anatomical socket levels (apex, mid region, and entrance), and the averaged values (average of apex and mid, and average of apex, mid, and entrance) were defined as used in the main analysis tables.

To reduce detection bias, DICOM exports and ROI files were coded with anonymized identifiers that concealed group allocation and time point. The assessor performing ROI preparation and ImageJ FracLac processing had no access to the randomization list and was blinded to treatment group during fractal computation.

Intra-observer and inter-observer reliability of fractal dimension measurements were assessed on a prespecified random sample of scans (recommended: at least 20 percent of participants, balanced across groups and time points). The primary assessor repeated the full ROI selection and fractal computation after a washout period of at least 2 weeks. A second independent assessor repeated the same workflow on the same sample. Reliability was quantified using the intraclass correlation coefficient (ICC) with 95% confidence intervals.

#### 2.6.3. Hierarchy of Endpoints

To address the multiplicity of outcomes and repeated measurements, a predefined hierarchy of endpoints was established. The primary endpoint of the study was postoperative pain intensity assessed using the 11-point visual analogue scale on postoperative day 3. The secondary endpoints included the following:

1. Postoperative pain intensity assessed on day 1 and day 7.
2. Postoperative swelling measured using linear facial measurements A, B, and C and the composite swelling index on days 1, 3, and 7.
3. Postoperative trismus assessed as change in maximal interincisal distance on days 1, 3, and 7.
4. Early wound healing assessed using the early healing index on day 7.
5. Fractal dimension of alveolar bone at 4 months after extraction compared with reference bone values.

Exploratory analyses included time-dependent within-group comparisons of clinical parameters and subgroup analyses of individual alveolar regions in fractal assessment.

### 2.7. Randomization

Patients were randomly allocated to the six study groups using an opaque sealed-envelope method. Prior to study initiation, allocation assignments corresponding to the six intervention arms in a 1:1:1:1:1:1 ratio were prepared and placed into sequentially numbered, identical, and opaque envelopes. The envelopes were sealed and stored in a secure location accessible only to a study team member not involved in outcome assessment.

#### Allocation Concealment and Blinding

Allocation concealment was ensured using an opaque, sequentially numbered, sealed envelope system. Each envelope contained a card indicating the assigned study group. Envelopes were identical, non transparent, and sealed with tamper-evident adhesive. They were stored in a locked cabinet accessible only to the research coordinator. Envelopes were opened sequentially and only after the patient had been deemed eligible and had provided written informed consent.

Implementation of the allocation sequence was performed by a research team member who was not involved in outcome assessment. The surgeon was informed of group allocation only after envelope opening immediately before surgery.

This trial was conducted as a single blind study. Participants were partially blinded in that they were not informed about the specific type of platelet concentrate used, but complete blinding was not feasible because venous blood collection and photobiomodulation procedures were apparent. The operating surgeon was not blinded due to the nature of the interventions. Postoperative clinical and radiological outcome assessments were performed by an investigator who was not involved in the surgical procedures and who remained blinded to group allocation throughout data collection and analysis.

### 2.8. Statistical Methods

#### 2.8.1. General Principles and Analysis Set

All statistical analyses were performed using IBM SPSS Statistics 29. The primary analysis followed an intention-to-treat principle, including all randomised participants with available outcome data. Longitudinal models were used because they provide valid inference under a missing-at-random assumption without requiring imputation. A per-protocol sensitivity analysis restricted to participants who completed all scheduled follow-ups was also performed to confirm the robustness of the key findings.

#### 2.8.2. Primary Endpoint Analysis

The primary endpoint was postoperative pain intensity (VAS, 0 to 10) on postoperative day 3. The primary comparison was the overall between-group difference across the six arms at day 3. This was assessed using a linear model with group as a fixed effect. If the overall test was significant, prespecified pairwise comparisons of control versus each active group were performed with multiplicity adjustment, as described below. The two-sided alpha level for the primary endpoint was 0.05.

#### 2.8.3. Repeated Measures Outcomes and Group × Time Interaction

Pain (VAS), swelling (composite swelling index and line-specific measures), and trismus (maximal interincisal distance change) were analysed using linear mixed-effects models with fixed effects for group (six levels), time (day 1, day 3, and day 7), and the group × time interaction, and a participant-specific random intercept to account for within-subject correlation. An unstructured covariance matrix was used when model convergence allowed; otherwise, a first-order autoregressive covariance structure was applied. The primary longitudinal inferential target was the group × time interaction. When the interaction was statistically significant, adjusted between-group contrasts at each time point were estimated from model-based marginal means. When the interaction was not significant, it was removed and the main-effects model was used to estimate an overall group effect averaged across time.

#### 2.8.4. Non-Normality and Model Diagnostics

Model residuals were inspected for severe deviations from normality and heteroscedasticity. If substantial departures were present, outcomes were analysed using a rank-based approach by fitting the mixed model to rank-transformed values as a sensitivity analysis. Conclusions were based on consistency between the primary model and sensitivity analyses.

#### 2.8.5. Single Time Point Outcomes

EHI (day 7) was analysed using an ordinal approach (Kruskal–Wallis test), with effect size reported as epsilon squared, followed by adjusted pairwise comparisons if the global test was significant. The proportion of complete wound closure was analysed using chi-square testing with effect size reported as Cramers V.

#### 2.8.6. CBCT Fractal Analysis

Fractal dimensions measured immediately after extraction and at 4 months were analysed using a two-time point linear mixed-effects model, including group, time, and group × time interaction, with a participant-specific random intercept. Because multiple fractal regions were analysed (apex, mid region, entrance, and prespecified averages), multiplicity control for fractal outcomes was applied using the Benjamini–Hochberg false discovery rate procedure within the family of fractal comparisons.

#### 2.8.7. Multiplicity Control

Multiplicity adjustment was predefined as follows. For the primary endpoint, the global six-group test used alpha 0.05. If significant, control versus each active group pairwise comparisons at day 3 were adjusted using the Holm procedure. For secondary longitudinal outcomes, family wise error was controlled within each outcome by applying the Holm procedure to post hoc pairwise comparisons that were triggered only by a significant group × time interaction (or significant main group effect when the interaction was removed). No unadjusted post hoc testing was performed.

#### 2.8.8. Effect Sizes and Confidence Intervals

For continuous outcomes, effect estimates were reported as model-based mean differences with 95% confidence intervals. For the primary endpoint, standardised effect sizes (Hedges g) were additionally reported for control versus each active group at day 3, with 95% confidence intervals derived from the corresponding model-based contrasts. For non-parametric comparisons, effect sizes were reported using appropriate rank-based measures, as stated above.

#### 2.8.9. Missing Data

Reasons for missing follow-up data were summarised by group. The primary mixed-model analyses used all available observations and therefore did not require imputation. As a sensitivity analysis, complete case per-protocol analyses were conducted and compared with the primary results.

## 3. Results

### 3.1. General Characteristics of Patients

**Figure 3:**
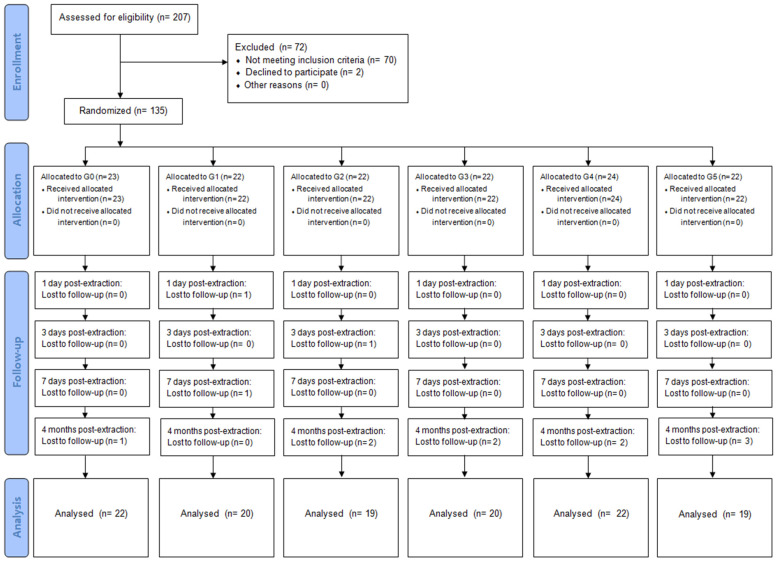
CONSORT 2010 flow diagram showing participant enrolment, allocation, follow-up, and analysis.

The study included 135 patients aged 18–40 years, while the total number of study participants who completed all follow-up visits was 122. The primary analysis followed an intention-to-treat principle using all available observations. A per-protocol sensitivity analysis was additionally performed among participants who completed all scheduled follow-up visits. Among the patients who completed the study, 26 were men and 96 were women. The mean age was 28.16 (SD = 3.73) years. Between August 2020 and September 2021, 135 partially or completely retained lower teeth were extracted at the outpatient dental surgery clinic of the University Dental Centre in Wroclaw. The average procedure time was 32.2 min. The number of participants is depicted in the flow diagram presented in Figure 3.

Baseline demographic and surgical characteristics were comparable across the six randomised groups (Table 2). No statistically significant between-group differences were observed for age, sex distribution, procedure duration, or extraction technique. These findings support adequate balance achieved through randomization.

### 3.2. Pain Assessment

Pain (VAS 0 to 10) measured at postoperative days 1, 3, and 7 was analysed using a linear mixed-effects model with fixed effects for group (six arms), time, and the group × time interaction, and a participant-specific random intercept. The group × time interaction was not statistically significant (Wald chi-square(10) = 11.56, *p* = 0.316), and the overall main effect of the group was not statistically significant (Wald chi-square(5) = 3.96, *p* = 0.555). A statistically significant main effect of time was observed (Wald chi-square(2) = 6.42, *p* = 0.040), indicating a change in pain over follow-up.

At postoperative day 3 (primary endpoint time point), model-based marginal means were: control 2.95, PBM 1.00, A-PRF 1.79, CGF 1.55, A-PRF + PBM 2.23, and CGF + PBM 1.68. Compared with control at day 3, PBM showed significantly lower pain (mean difference −1.95 VAS points; 95 percent CI −3.19 to −0.72; Holm-adjusted *p* = 0.010; Hedges g = 0.86). Differences versus the control at day 3 for CGF (mean difference −1.40; 95 percent CI −2.64 to −0.17; Holm-adjusted *p* = 0.105) and CGF + PBM (mean difference −1.27; 95 percent CI −2.53 to −0.01; Holm-adjusted *p* = 0.142) did not remain statistically significant after multiplicity adjustment.

Non-parametric Kruskal–Wallis and Wilcoxon analyses are reported as sensitivity analyses below (Table 3).

A Kruskal–Wallis H-test analysis was performed to compare pain experienced after treatment among patients in each tissue engineering and photobiomodulation group.

The analysis revealed a statistically significant and average difference in pain scores three days after surgery. Further analysis of paired comparisons demonstrated that patients who underwent mandibular third molar extraction without tissue engineering methods or photobiomodulation experienced more pain three days after surgery than patients who received photobiomodulation (*p* < 0.05). Subsequently, we examined how pain scores after mandibular third molar extraction evolved over time for the different tissue bioengineering and photobiomodulation methods. To this end, a Wilcoxon analysis was conducted, and the results are presented in Table 4.

The analysis demonstrated that in the group which did not undergo tissue engineering or photobiomodulation methods following mandibular third molar extraction, there was a deterioration of pain 3 days after surgery (M = 2.95; SD = 2.65) in comparison with day 1 after surgery (M = 1.91; SD = 1.95), followed by a reduction in pain 7 days after surgery (M = 1.86; SD = 2.34) to a level similar to the pain on day 1 after surgery. The value of the effect strength index r indicates the average magnitude of these differences.

In patients in the G2 group, after extraction of the mandibular third molar was treated with A-PRF+, postoperative pain did not subside until day 7 postoperatively (M = 1.00; SD = 1.45) compared with day 1 post-extraction (M = 2.11; SD = 1.94). The value of the effect strength index r indicates the average size of these differences. Additionally, at the level of statistical trend, pain 3 days after surgery was at a moderately higher level than perceived pain 7 days after surgery. For the other tissue engineering and photobiomodulation methods, no statistically significant differences were found between the individual measures of pain assessment after mandibular third molar extraction on day 1 after surgery, day 3 after surgery and day 7 after surgery.

### 3.3. Oedema

Composite swelling (mean change from baseline across lines A, B, and C) measured at days 1, 3, and 7 was analysed using a linear mixed-effects model. The group × time interaction was not statistically significant (Wald chi-square(10) = 7.79, *p* = 0.650), and the overall main effect of group was not statistically significant (Wald chi-square(5) = 5.13, *p* = 0.401). A statistically significant main effect of time was observed (Wald chi-square(2) = 22.37, *p* = 0.000014), consistent with the expected postoperative swelling trajectory.

Line-specific swelling results and non-parametric analyses are provided as sensitivity analyses below. To this end, a Kruskal–Wallis H analysis was conducted, and the outcomes are delineated in Table 5.

The subsequent analysis employed the Mann–Whitney U test, which revealed that on the first day following mandibular third molar extraction, patients who underwent photobiomodulation exhibited a reduced degree of B-line swelling in comparison to the other patients (*p* < 0.05). No statistically significant differences were observed among the remaining study groups. Furthermore, the study investigated whether tissue engineering methods and photobiomodulation independently influenced the extent of swelling post-mandibular third molar extraction, independent of its location. To this end, an index of the overall swelling magnitude was calculated based on the mean swelling of lines A, B and C, followed by Kruskal–Wallis H analysis (Table 6).

**Figure 4:**
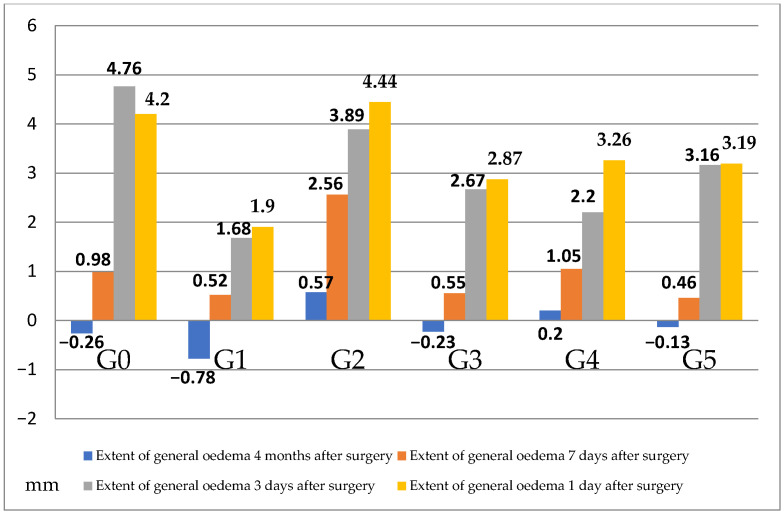
Distribution of the mean extent of overall oedema after extraction for the different groups of tissue engineering and photobiomodulation methods used.

The analysis performed revealed no statistically significant differences in the magnitude of postoperative swelling between the tissue engineering and photobiomodulation methods. This finding corroborates earlier conclusions about the absence of differences between the methods in the extent of swelling of individual lines. However, given the statistical significance of the observed differences in the magnitude of overall swelling 1 day after extraction, an additional Mann–Whitney U test analysis was conducted to compare the study groups in terms of the magnitude of swelling 1 day after surgery. The analysis yielded a statistically significant result (Z = −2.90; *p* = 0.004), indicating that, on average, the magnitude of overall swelling one day after extraction was greater in subjects without tissue engineering or photobiomodulation methods than in subjects who received photobiomodulation after mandibular third molar extraction. The distribution of the averages is illustrated in Figure 4.

### 3.4. Trismus

Trismus (change in maximal interincisal distance from baseline) measured at days 1, 3, and 7 was analysed using a linear mixed-effects model. The group × time interaction did not reach statistical significance (Wald chi-square(10) = 16.65, *p* = 0.083), and the overall main effect of group was not statistically significant (Wald chi-square(5) = 1.31, *p* = 0.934). A statistically significant time effect was observed (Wald chi-square(2) = 6.88, *p* = 0.032), indicating improvement over the follow-up period. Non-parametric within-group comparisons are presented as sensitivity analysis below.

In order to test the hypothesis regarding the effect of the type of tissue engineering and photobiomodulation method used on the improvement in the postoperative maxillary dilation range, a Kruskal–Wallis H analysis was performed (Table 7).

The analysis demonstrated a high degree of similarity in the extent of changes observed among the various treatment methods employed following third molar extraction. The patient groups under study exhibited no significant disparities in the post-extraction extent of jaw dilation alterations across all phases of treatment: day 1 post-extraction, day 3 post-extraction, day 7 post-extraction, and 4 months post-extraction.

In the subsequent stage of the analysis, the alterations in post-extraction maxillofacial pressure over time were investigated across the various patient groups. To this end, a Wilcoxon analysis was conducted, and the outcomes are presented in Table 8.

For the majority of groups, with the exception of patients who underwent extraction of the mandibular third molar with photobiomodulation, the reduction in mandibular compression progressed gradually from day 7 after surgery until the fourth month after extraction. A subsequent comparison of the strength of the difference effect between the groups revealed that the most significant decrease in jawbone density on day 7 occurred among patients who received a combination of A-PRF+ and CGF alveolar supply along with the application of photobiomodulation, and over the period from day 7 to the fourth month post-extraction in the group in which the A-PRF+ alveolar provision was used, the change in maxillary compression appeared to be less than that of CGF, which may indicate a slightly faster decrease in maxillary compression in the group in which the A-PRF+ alveolar provision was used.

### 3.5. Hard Tissue Reparation

The investigation then proceeded to examine whether the fractal dimensions of the alveolus exhibited variations four months following extraction, in comparison to a hypothetical reference value derived from the mean fractal dimensions of the reference bone of the subjects (M = 1.46). To this end, a one-sample Student’s *t* analysis was conducted, and the outcomes are presented in Table 9.

**Figure 5:**
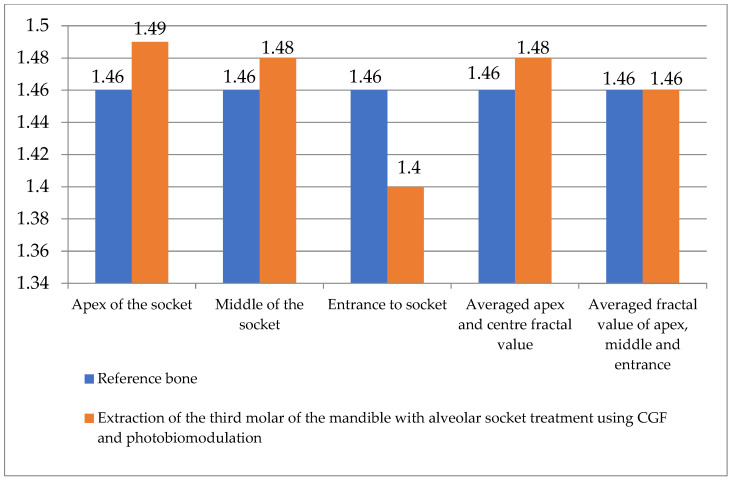
Differences between fractal values of reference bone and actual alveolar measurement in group 5 (G5).

The CGF + PBM group demonstrated numerically lower values for selected outcomes; however, not all between-group differences reached statistical significance. The alveolar crest, alveolar region, averaged fractal value of the crest and centre, and averaged fractal value of the crest, centre and alveolar entry exhibited a fractal dimension that was analogous to that of the reference bone (M = 1.46). However, the alveolar entry exhibited a divergent fractal dimension from the reference bone, suggesting incomplete ossification. The disparities between the hypothetical reference bone and the actual fractal measurements, collected four months post-extraction, for the group that received CGF alveolar supply and photobiomodulation therapy, are delineated in Figure 5.

The findings revealed that the alveolar entry and the averaged fractal value of the apex, centre and alveolar entry exhibited a reduced fractal dimension in comparison to the reference bone. This indicated that bone reconstruction exhibited a weaker mineralization and microarchitecture than the reference bone. The extraction of the mandibular third molar without the utilisation of tissue engineering or photobiomodulation methods yielded the least optimal results in terms of bone reconstruction. Statistically significant differences were identified between all alveolar areas examined (including averaged fractal values), with the exception of the alveolar crest. This indicates that only in the case of the alveolar ridge was there a complete fusion of bone tissue. Excluding the applications of tissue engineering and photobiomodulation, a comparison was made between the hypothetical fractal value of the reference bone and the individual alveolar areas. This analysis revealed that the alveolar peak and the averaged fractal value of the alveolar peak and centre exhibited a high degree of similarity to the fractal dimension of the reference bone. This finding suggests that alveolar healing occurs in these areas.

### 3.6. Soft Tissue Reparation

Subsequently, an investigation was conducted to ascertain whether the occurrence of wound closure, as determined on the seventh day following mandibular third molar extraction, was contingent upon the tissue engineering and photobiomodulation methodology employed. To this end, Kruskal–Wallis H analysis was conducted, and the outcomes are presented in Table 10.

The statistical significance of the analysis performed was insufficient to identify statistically significant differences between the compared groups, thereby demonstrating that the degree of wound closure following mandibular third molar extraction is independent of the tissue engineering and photobiomodulation methods employed. Furthermore, an investigation was conducted to ascertain whether there was a discrepancy in the incidence of complete and incomplete wound closure according to the EHI between the tissue engineering and photobiomodulation methods used. To this end, a chi-square independence analysis was conducted, and the results are presented in Table 11.

The results of the χ^2^ analysis demonstrate a comparable distribution of patients who have undergone complete wound closure following third molar extraction. The majority of subjects within each study group did not yet have full wound closure on the seventh day following the extraction procedure.

Regarding the predefined hypotheses, the hypothesis that photobiomodulation combined with CGFs results in reduced postoperative pain and improved bone remodelling was partially confirmed. The hypothesis that patients not receiving regenerative methods would experience the greatest postoperative swelling was confirmed for the early postoperative period. No significant differences were observed between groups with respect to postoperative trismus, and therefore this hypothesis was not confirmed.

### 3.7. Reliability of Fractal Dimension Measurements

Intra-observer and inter-observer reliability of fractal dimension measurements were assessed using intraclass correlation coefficients (ICCs) based on a two-way random-effects model with absolute agreement. A prespecified random sample representing at least 20 percent of participants, balanced across groups and time points, was re-analysed.

Intra-observer reliability demonstrated excellent agreement across all socket levels. The ICC values were

Apex region: ICC = 0.94 (95 percent CI 0.88 to 0.97);
Mid-alveolar region: ICC = 0.96 (95 percent CI 0.91 to 0.98);
Socket entrance: ICC = 0.92 (95 percent CI 0.85 to 0.96).

Inter-observer reliability was similarly high:

Apex region: ICC = 0.91 (95 percent CI 0.82 to 0.95);
Mid-alveolar region: ICC = 0.93 (95 percent CI 0.86 to 0.97);
Socket entrance: ICC = 0.89 (95 percent CI 0.79 to 0.94).

All ICC values indicated good to excellent reproducibility of ROI selection and fractal computation.

### 3.8. Postoperative Adverse Events and Rescue Analgesic Use

Postoperative adverse events were recorded prospectively during follow-up. The incidence of complications was low across all groups. Alveolitis occurred in seven cases overall: two in the control group, one in the PBM group, one in the A-PRF+ group, two in the CGF group, one in the A-PRF+ + PBM group, and none in the CGF + PBM group. Infection requiring antibiotic therapy was recorded in four cases, occurring exclusively in the PBM (*n* = 2) and A-PRF+ (*n* = 2) groups. No infections requiring antibiotics were observed in the control, CGF, A-PRF+ + PBM, or CGF + PBM groups. No cases of postoperative bleeding requiring additional surgical intervention were recorded. No persistent neurological symptoms were observed in any group. Given the low absolute number of events, no statistically meaningful between-group comparison was performed. Detailed data are presented in Table 12.

Rescue analgesic use (paracetamol) during the first 72 h was documented using standardised medication diaries. The proportion of participants requiring rescue analgesia did not differ significantly between groups. The median number of rescue doses was low across all groups, and the inclusion of rescue analgesic use as a covariate in sensitivity analyses did not alter the primary endpoint results.

## 4. Discussion

The interpretation of the results should consider that postoperative pain on day 3 constituted the primary endpoint, while other clinical and radiological parameters were secondary or exploratory outcomes. The present study evaluated the effects of APCs and PBM on the healing process following mandibular third molar extraction. The present findings suggest that selected interventions, particularly photobiomodulation and CGFs combined with photobiomodulation, were associated with improvements in specific postoperative outcomes, most notably, pain at postoperative day 3 and selected fractal dimension parameters at 4 months. However, no statistically significant intergroup differences were observed for trismus, overall facial swelling, or EHI scores, and therefore the results should not be interpreted as demonstrating a uniform effect across all dimensions of postoperative recovery. One of the key observations was the effect of photobiomodulation on postoperative oedema. Patients who underwent PBM therapy exhibited significantly lower oedema values compared to G0, particularly in the early postoperative period. The statistical analysis confirmed a significant reduction in oedema one day after surgery among patients who received PBM, supporting the hypothesis that laser therapy has an immediate anti-inflammatory effect [29]. Postoperative oedema, and, more specifically horizontal swelling determined by line B, showed lower values for patients undergoing LLLT on postoperative day 1 relative to the other methods studied. This relationship, although remaining at the level of a statistical trend, was evident in the generalised oedema (understood as the average of all three measurement lines in a given measurement) on postoperative day 1 and showed higher values in patients without regenerative methods compared to patients who received LLLT. In contrast, the most significant reduction in oedema that had already developed during the initial seven-day period was observed in the A-PRF+ group, particularly in the group that received CGFs and photobiomodulation. Whilst all groups demonstrated a natural decline in pain, patients who received PBM (G1, G4, and G5) exhibited significantly lower pain scores on day 3 after surgery in comparison to those who did not receive laser therapy. This finding is consistent with previous studies on PBM, which suggests that low-level laser therapy can modulate inflammatory mediators and reduce nociceptive responses. Moreover, the study did not prove the hypothesis that the CGF dressing, when used in combination with LLLT, would reduce pain. However, it was demonstrated that post-extraction alveoli irradiated with LLLT in a monotherapeutic regimen experienced less pain than G0.

Many studies confirm the results obtained by the authors of this study [4,18,30,31,32]. However, a meta-analysis published by Lacerda-Santos et al. found only little or very little evidence for the effect of PBM in controlling pain, oedema and trismus after third molar extractions [33]. The radiation emitted by the low-power laser is a monochromatic beam of light, which exerts thermal photobiological effects without destroying the surrounding tissue. The wavelength of the beam determines its capacity to penetrate structures. PBM therapy engenders cellular changes that promote cell viability, proliferation, and tissue healing [34]. The therapy under discussion is based on a photochemical mechanism whereby energy is transferred to intracellular mitochondrial chromophores. Such molecules are capable of light absorption, and examples include endogenous porphyrins and certain components of the respiratory chain, such as cytochrome-C oxidase. These molecules can transfer the absorbed laser energy to mitochondria, where it is converted into metabolic energy through the respiratory chain, producing adenosine triphosphate (ATP). The absorption of light by respiratory chain components results in a short-term activation of the respiratory chain itself and the oxidation of nicotinamide adenine dinucleotide (NADH), leading to alterations in both mitochondrial and cytoplasmic redox states. The induction of various biological changes, including oedema, has been demonstrated to contribute to the alleviation of postoperative symptoms [35].

The combination of CGF and photobiomodulation demonstrated favourable outcomes in selected parameters, particularly postoperative pain at day 3 and fractal dimension values in the mid-alveolar region at 4 months. While these findings may suggest a potential additive or complementary biological effect, the evidence does not consistently demonstrate statistically significant superiority across all evaluated endpoints. For several clinical parameters, including trismus, overall swelling, and EHI scores, differences between groups were not statistically significant. Therefore, the concept of a synergistic interaction between CGF and photobiomodulation should be interpreted cautiously and considered hypothesis-generating rather than definitively established. Patients in groups G2 and G3 (A-PRF+ and CGF, respectively) exhibited enhanced wound closure, which aligns with previous research indicating that growth factors within APCs enhance fibroblast activity and angiogenesis. A number of recent systematic reviews have identified the potential for platelets to play a critical role in tissue regeneration due to their function as repositories of growth factors that are essential for such regenerative procedures [36,37,38]. The most significant feature of the CGF and PRF is its consistency, which allows it to act as a growth factor repository and natural scaffolding. In essence, the CGF represents an enhanced version of the PRF, characterised by a more robust fibrin matrix and elevated levels of cytokines and growth factors [37]. It has been demonstrated that these growth factors attract undifferentiated mesenchymal cells to the site of injury, thereby facilitating processes such as angiogenesis, chemotaxis and cell proliferation [39].

The investigation revealed that none of the therapeutic modalities examined, including CGF with LLLT, exhibited a significant impact on the reduction in postoperative jawbone formation. However, it was observed that the most substantial decrease in the pre-existing jawbone, recorded between days 1 and 7, was achieved when CGF with LLLT and A-PRF+ were employed. Contrary results regarding trismus after extraction of the third lower molars were found by the authors of other studies [40,41,42].

From a radiological perspective, fractal analysis of bone regeneration demonstrated that patients treated with CGF and PBM exhibited the highest bone density at the 4-month follow-up. The statistical comparison of fractal dimensions indicated that CGF, particularly when combined with PBM, accelerates alveolar bone remodelling, making it a promising approach for enhancing post-extraction bone healing. Furthermore, the combination of CGF and PBM (G5) resulted in the most pronounced improvement in early healing outcomes, suggesting a potential synergistic effect between platelet-derived cytokines and laser biostimulation. PBM therapy, otherwise referred to as low-level light therapy (LLLT), has been shown to increase collagen production and contribute to connective tissue stability by stimulating the proliferation and maturation of fibroblasts. The biostimulating effect of LLLT on bone is thought to be achieved through expanding the organic bone matrix, increasing the mitotic capacity of osteoblasts, stimulating their proliferation and differentiation, and increasing the quantity and activity of differentiated osteoblasts [43,44,45,46,47]. The Akkaya and Toptaş study established that, in the context of early bone regeneration in the extraction socket, the application of PRF and diode laser therapy as standalone interventions is ineffective. However, the concurrent administration of PRF and diode laser therapy has been demonstrated to exert a statistically significant impact on this process [48]. Chou et al. (2025) demonstrated in their study that PRF and PBM enhance bone regeneration in preosteoblasts. They further showed that PRF and PBM improve cell migration, which is crucial for bone healing, and that the synergistic application of PRF and PBM increases calcium deposition, especially after 7 and 14 days of treatment [49]. A very important factor in the results of this study was the fact that none of the patients received antibiotics after the procedure. This was due to the restrictive exclusion criteria. Studies show that postoperative antibiotic therapy significantly affects the parameters that were assessed in this study [50,51,52]. Due to the absence of antibiotics, the results were not distorted at this level. A new direction in research is currently the use of APCs as carriers for antibiotics, which could translate into maintaining the advantages of reducing postoperative symptoms while simultaneously reducing antibiotic resistance associated with systemic antibiotic use [15,53]. A study by Bilginayla et al. showed that the use of PRF with the addition of antibiotics gives better results in terms of pain, jaw clenching, and swelling compared to the use of PRF alone [54].

Several limitations of this study should be considered. The exclusion of exceptionally difficult mandibular third molar impactions may limit the applicability of the findings to more complex surgical cases. Due to the nature of the interventions, complete blinding of participants and the operating surgeon was not feasible; however, postoperative outcomes were assessed by a blinded investigator. The lack of a power metre during diode laser application may have resulted in minor variability in delivered laser energy. In addition, preoperative blood tests were not performed to determine platelet count prior to the preparation of autologous platelet concentrates. Although the sample size was supported by an a priori rationale based on the primary endpoint, the six-arm design reduced the number of participants per group and therefore limited statistical power for detecting small-to-moderate differences in secondary outcomes and for identifying group-by-time interaction effects. In addition, the evaluation of multiple outcomes and repeated assessments increases multiplicity, and non-significant findings for secondary endpoints should be interpreted cautiously as they may reflect limited power rather than true absence of effects. An additional limitation of this study is the predominance of female participants in the study population. Although sex distribution was comparable across randomised groups, the overall gender imbalance may limit external validity. Biological sex may influence inflammatory responses, pain perception, and wound healing dynamics, and therefore the findings may not be fully generalizable to a balanced or predominantly male population. Finally, the single-centre design and relatively short follow-up period limit the evaluation of long-term outcomes.

## 5. Conclusions

The results of this randomised controlled trial indicate that both autologous platelet concentrates and photobiomodulation may support the healing process following mandibular third molar extraction. Photobiomodulation was associated with a reduction in early postoperative pain and swelling, while the use of platelet concentrates contributed to favourable soft tissue healing and bone remodelling. The combined application of CGF and photobiomodulation showed consistent trends toward improved healing outcomes; however, not all evaluated parameters demonstrated statistically significant differences between groups. Therefore, these regenerative approaches should be considered as supportive rather than definitive interventions, and further well-designed studies are required to optimise clinical protocols and confirm their long-term effects.

## Figures and Tables

**Table 1:** Inclusion and exclusion criteria for patients.

Inclusion Criteria	Exclusion Criteria
- Patients aged 18–40 - Presence of a partially or completely retained third lower molar - Consent to participate in the study - No antibiotics taken in the last month before extraction	- Smoking - General diseases - Metabolic disorders - Pregnancy - Alcoholism - Plaque index (PI) > 25% - Exceptionally difficult position of a retained tooth - Extraction time exceeding 60 min

**Table 2:** Baseline demographic and surgical characteristics by randomised group.

Characteristic	**G0 Control** **(*n* = 22)**	**G1 PBM** **(*n* = 20)**	**G2 A-PRF+** **(*n* = 19)**	**G3 CGF** **(*n* = 20)**	**G4 A-PRF+ + PBM (*n* = 22)**	**G5 CGF + PBM (*n* = 19)**	***p* Value**
Age (years), mean ± SD	28 ± 3.6	28.6 ± 3.9	27.7 ± 3.5	28.4 ± 3.8	28.1 ± 3.7	28.2 ± 3.6	0.982
Female, *n* (%)	11 (50%)	13 (65%)	11 (57.9%)	12 (60%)	11 (50%)	10 (52.6%)	0.239
Procedure duration (min), mean ± SD	32.55 ± 15.32	26.70 ± 14.64	29.26 ± 13.02	35.75 ± 14.16	35.36 ± 20.20	33.21 ± 11.41	
Resection required, *n* (%)	11 (50%)	11 (55.0%)	9 (47.4%)	11 (55%)	13 (59.1%)	9 (47.4%)	

**Table 3:** Results of Kruskal–Wallis H-test analysis comparing patients from each group of tissue engineering and photobiomodulation methods in terms of pain experienced after the procedure.

Variable (VAS Score)	Group	Mean Rank	M	Me	SD	H(df)	*p*	η^2^
Pain assessment 1 day after surgery	G0	64.09	1.91	2.00	1.95	7.67	0.176	0.02
	G1	48.58	1.10	1.00	1.52			
	G2	69.68	2.11	2.00	1.94			
	G3	59.03	1.45	1.00	1.43			
	G4	72.95	2.05	1.50	1.50			
	G5	53.26	1.42	1.00	2.12			
Pain assessment 3 days after surgery	G0	76.84	2.95	2.00	2.65	11.23	0.047	0.05
	G1	44.33	1.00	0.00	1.65			
	G2	62.97	1.79	2.00	1.84			
	G3	55.28	1.55	1.00	2.33			
	G4	68.73	2.23	2.00	2.16			
	G5	58.53	1.68	1.00	2.11			
Pain assessment 7 days after surgery	G0	66.73	1.86	1.00	2.34	1.76	0.882	<0.01
	G1	62.05	1.60	0.50	2.14			
	G2	55.13	1.00	0.00	1.45			
	G3	59.90	1.65	0.50	2.60			
	G4	65.39	1.95	1.00	2.72			
	G5	58.42	1.58	0.00	2.48			

M—mean; Me—median; SD—standard deviation; H(df)—value of test statistic and degrees of freedom; *p*—statistical significance; η^2^—effect strength index. The observed effect sizes ranged from small to moderate, indicating that the magnitude of the differences between groups, although statistically significant in selected outcomes, was limited.

**Table 4:** Results of Wilcoxon analysis testing for differences between pain complaints at different days after extraction by group of tissue bioengineering and photobiomodulation methods used.

Group		*z*	*p*	r
G0	Pain assessment 1 day after surgery vs. 3 days after surgery	−2.01	0.045	0.30
	Pain assessment 1 day after surgery vs. 7 days after surgery	−0.29	0.074	0.04
	Pain assessment 3 days after surgery vs. 7 days after surgery	−2.27	0.023	0.34
G1	Pain assessment 1 day after surgery vs. 3 days after surgery	−0.44	0.659	0.07
	Pain assessment 1 day after surgery vs. 7 days after surgery	−0.78	0.438	0.12
	Pain assessment 3 days after surgery vs. 7 days after surgery	−0.9	0.370	0.14
G2	Pain assessment 1 day after surgery vs. 3 days after surgery	−0.52	0.605	0.08
	Pain assessment 1 day after surgery vs. 7 days after surgery	−1.98	0.048	0.32
	Pain assessment 3 days after surgery vs. 7 days after surgery	−1.90	0.057	0.31
G3	Pain assessment 1 day after surgery vs. 3 days after surgery	−0.39	0.700	0.06
	Pain assessment 1 day after surgery vs. 7 days after surgery	−0.4	0.688	0.06
	Pain assessment 3 days after surgery vs. 7 days after surgery	−0.89	0.373	0.14
G4	Pain assessment 1 day after surgery vs. 3 days after surgery	−0.22	0.830	0.03
	Pain assessment 1 day after surgery vs. 7 days after surgery	−0.38	0.701	0.06
	Pain assessment 3 days after surgery vs. 7 days after surgery	−0.60	0.549	0.09
G5	Pain assessment 1 day after surgery vs. 3 days after surgery	−0.49	0.622	0.08
	Pain assessment 1 day after surgery vs. 7 days after surgery	−0.14	0.886	0.02
	Pain assessment 3 days after surgery vs. 7 days after surgery	−0.46	0.644	0.07

*z*—value of test statistic; *p*—statistical significance; r—effect strength index.

**Table 5:** Results of the Kruskal–Wallis H analysis comparing the extent of oedema in relation to time elapsed.

Variable		Group	Mean Rank	M (mm)	Me (mm)	SD (mm)	H(df)	*p*	η^2^
Extent of oedema 1 day after surgery	Oedema line A	G0	75.59	4.64	5.00	3.79	7.92	0.161	0.03
		G1	55.90	2.50	0.00	4.44			
		G2	67.84	4.58	5.00	6.71			
		G3	50.58	1.75	0.00	3.73			
		G4	58.30	2.50	0.00	2.99			
		G5	59.95	2.79	0.00	3.43			
	Oedema line B	G0	67.61	3.68	4.00	2.15	11.73	0.039	0.07
		G1	38.43	1.45	0.50	3.02			
		G2	63.45	4.16	3.00	4.72			
		G3	61.00	3.40	3.50	3.05			
		G4	71.80	3.91	5.00	2.84			
		G5	65.37	3,58	3.00	2.50			
	Oedema line C	G0	71.66	4,27	4.50	2.99	6.98	0.222	0.03
		G1	44.53	1.75	1.50	2.71			
		G2	65.79	4.58	5.00	6.97			
		G3	63.73	3.45	3.50	2.98			
		G4	61.61	3.36	4.00	2.92			
		G5	60.84	3.21	3.00	2.64			
Extent of oedema 3 days after surgery	Oedema line A	G0	71.05	4.41	5.00	6.80	0.511	0.357	0.01
		G1	59.58	2.50	1.00	6.74			
		G2	59.61	3.53	0.00	10.78			
		G3	65.60	3.60	5.00	3.44			
		G4	48.34	0.50	0.00	6.21			
		G5	65.29	2.95	5.00	7.41			
	Oedema line B	G0	72.73	4/45	5.00	5.33	6.40	0.269	0.02
		G1	49.50	0.25	0.00	6.79			
		G2	60.16	2.84	1.00	6.80			
		G3	59.98	1.95	3.00	6.82			
		G4	69.27	3.45	5.00	5.52			
		G5	55.08	1.47	0.00	6.06			
	Oedema line C	G0	72.27	5.41	5.50	4.89	5.19	0.394	0.01
		G1	54.48	2.30	4.00	6.09			
		G2	62.61	5.32	5.00	8.96			
		G3	61.43	2.45	4.50	9.98			
		G4	51.39	2.64	3.00	9.51			
		G5	67.11	5.05	3.00	5.18			
Extent of oedema 7 days after surgery	Oedema line A	G0	71.34	1.73	0.00	2.37	4.30	0.506	<0.01
		G1	61.95	1.25	0.00	3.93			
		G2	61.74	2.11	0.00	6.08			
		G3	55.95	0.65	0.00	3.00			
		G4	61.41	0.73	0.00	2.73			
		G5	55.34	0.26	0.00	2.62			
	Oedema line B	G0	62.61	0.77	0.00	2.11	5.16	0.396	0.01
		G1	53.70	0.25	0.00	2.34			
		G2	68.87	2.58	0.00	5.79			
		G3	54.93	0.45	0.00	3.50			
		G4	71.30	1.64	0.50	2.94			
		G5	56.63	0.63	0.00	2.52			
	Oedema line C	G0	61.84	0.45	0.00	2.32	1.77	0.880	0.02
		G1	55.50	0.05	0.00	2.56			
		G2	66.55	3.00	0.00	7.70			
		G3	58.68	0.55	0.00	2.70			
		G4	66.77	0.77	0.00	3.66			
		G5	59.24	0.47	0.00	2.09			
Extent of oedema 4 months after surgery	Oedema line A	G0	59.45	0.00	0.00	2.18	2.72	0.743	0.01
		G1	51.08	−0.67	0.00	2.38			
		G2	59.83	0.11	0.00	1.41			
		G3	59.73	0.25	0.00	3.43			
		G4	61.82	0.23	0.00	2.51			
		G5	64.56	0.33	0.00	2.61			
	Oedema line B	G0	56.75	−0.27	0.00	1.96	5.45	0.364	0.01
		G1	49.97	−1.00	0.00	2.81			
		G2	67.53	0.78	0.00	3.10			
		G3	53.43	−0.50	0.00	2.50			
		G4	69.70	0.73	0.00	3.09			
		G5	58.64	−0.22	0.00	2.46			
	Oedema line C	G0	58.82	−0.50	0.00	2.20	0.95	0.967	0.03
		G1	57.28	−0.67	0.00	2.57			
		G2	66.39	0.83	0.00	3.67			
		G3	59.08	−0.45	0.00	1.91			
		G4	58.20	−0.36	0.00	2.38			
		G5	57.72	−0.50	0.00	3.20			

M—mean; Me—median; SD—standard deviation; H(df)—value of test statistic and degrees of freedom; *p*—statistical significance; η^2^—effect strength index.

**Table 6:** Results of the Kruskal–Wallis H analysis comparing the overall extent of oedema.

Variable	Group	Mean Rank	M (mm)	Me (mm)	SD (mm)	H(df)	*p*	η^2^
Extent of general oedema 1 day after surgery	G0	75.98	4.20	4.17	2.30	9.80	0.081	0.05
	G1	43.93	1.90	1.00	2.80			
	G2	66.82	4.44	3.67	5.66			
	G3	55.55	2.87	3.00	2.64			
	G4	64.09	3.26	3.50	2.28			
	G5	61.18	3.19	3.33	2.18			
Extent of general oedema 3 days after surgery	G0	76.61	4.76	5.67	4.62	5.75	0.311	0.02
	G1	53.00	1.68	1.17	5.09			
	G2	60.11	3.89	2.33	8.16			
	G3	60.45	2.67	2.17	5.16			
	G4	56.07	2.20	1.50	5.44			
	G5	61.74	3.16	1.00	5.35			
Extent of general oedema 7 days after surgery	G0	66.36	0.98	0.83	1.81	2.45	0.785	0.01
	G1	58.38	0.52	0.00	2.28			
	G2	66.87	2.56	0.00	6.35			
	G3	56.80	0.55	0.17	2.20			
	G4	65.34	1.05	0.83	2.26			
	G5	54.29	0.46	0.00	1.93			
Extent of general oedema 4 months after surgery	G0	58.09	−0.26	0.00	1.39	4.14	0.529	<0.01
	G1	48.61	−0.78	−0.50	1.77			
	G2	69.78	−0.57	0.00	1.88			
	G3	58.33	−0.23	0.00	2.03			
	G4	64.59	0.20	0.00	1.93			
	G5	56.92	−0.13	−0.17	1.97			

M—mean; Me—median; SD—standard deviation; H(df)—value of test statistic and degrees of freedom; *p*—statistical significance; η^2^—effect strength index.

**Table 7:** Results of Kruskal–Wallis H analysis of variance testing the relationship of postoperative changes in jaw dilation range to the use of tissue engineering and photobiomodulation methods.

Variable	Group	Mean Rank	M	Me	SD	H(df)	*p*	η^2^
Change in range of maxillary dilation—1 day after extraction	G0	58.41	11.18	12.00	8.15	1.12	0.952	0.47
	G1	61.10	11.80	5.50	9.77			
	G2	56.87	11.21	8.00	11.45			
	G3	63.05	12.30	12.50	8.34			
	G4	57.25	13.86	16.50	9.07			
	G5	61.84	11.95	9.00	9.62			
Change in range of maxillary dilation—3 days after extraction	G0	61.93	12.32	10.00	9.18	3.62	0.606	<0.01
	G1	67.47	14.30	13.50	11.07			
	G2	51.76	10.00	7.00	10.88			
	G3	69.15	13.90	14.50	8.54			
	G4	62.66	12.86	10.00	10.78			
	G5	55.05	10.68	7.00	9.45			
Change in range of maxillary dilation—7 days after extraction	G0	72.45	8.91	9.50	7.22	6.02	0.305	<0.01
	G1	67.53	9.05	5.00	10.01			
	G2	51.55	5.89	1.00	8.43			
	G3	62.73	5.95	6.00	5.62			
	G4	61.86	7.36	5.00	9.08			
	G5	50.71	4.84	3.00	6.38			
Change in range of maxillary dilation—4 months after extraction	G0	68.66	0.00	0.00	1.35	2.74	0.739	0.06
	G1	54.97	−0.61	−1.00	1.65			
	G2	59.03	−0.39	0.00	2.99			
	G3	62.33	−0.60	0.00	2.26			
	G4	54.52	−0.73	−0.50	1.78			
	G5	56.25	−0.50	−1.00	1.95			

M—mean; Me—median; SD—standard deviation; H(df)—value of test statistic and degrees of freedom; *p*—statistical significance; η^2^—effect strength index.

**Table 8:** Results of Wilcoxon analysis examining the time dependence of changes in the jaw dilation range.

Group	Variable	*z*	*p*	r
G0	Pain assessment 1 day after surgery vs. 3 days after surgery	−1.17	0.242	0.18
	Pain assessment 1 day after surgery vs. 7 days after surgery	−2.6	0.009	0.39
	Pain assessment 1 day after surgery vs. 4 months after surgery	−4.02	<0.001	0.61
	Pain assessment 3 days after surgery vs. 7 days after surgery	−3.14	0.002	0.47
	Pain assessment 3 days after surgery vs. 4 months after surgery	−4.02	<0.001	0.61
	Pain assessment 7 days after surgery vs. 4 months after surgery	−3.85	<0.001	0.58
G1	Pain assessment 1 day after surgery vs. 3 days after surgery	−1.59	0.111	0.25
	Pain assessment 1 day after surgery vs. 7 days after surgery	−1.23	0.218	0.19
	Pain assessment 1 day after surgery vs. 4 months after surgery	−3.63	<0.001	0.61
	Pain assessment 3 days after surgery vs. 7 days after surgery	−2.87	0.004	0.45
	Pain assessment 3 days after surgery vs. 4 months after surgery	−3.62	<0.001	0.60
	Pain assessment 7 days after surgery vs. 4 months after surgery	−3.36	<0.001	0.56
G2	Pain assessment 1 day after surgery vs. 3 days after surgery	−0.55	0.585	0.09
	Pain assessment 1 day after surgery vs. 7 days after surgery	−3.07	0.002	0.50
	Pain assessment 1 day after surgery vs. 4 months after surgery	−3.39	<0.001	0.57
	Pain assessment 3 days after surgery vs. 7 days after surgery	−3.23	0.001	0.52
	Pain assessment 3 days after surgery vs. 4 months after surgery	−3.64	<0.001	0.61
	Pain assessment 7 days after surgery vs. 4 months after surgery	−2.87	0.004	0.48
G3	Pain assessment 1 day after surgery vs. 3 days after surgery	−0.83	0.406	0.13
	Pain assessment 1 day after surgery vs. 7 days after surgery	−3.03	0.002	0.48
	Pain assessment 1 day after surgery vs. 4 months after surgery	−3.83	<0.001	0.61
	Pain assessment 3 days after surgery vs. 7 days after surgery	−3.58	<0.001	0.57
	Pain assessment 3 days after surgery vs. 4 months after surgery	−3.83	<0.001	0.61
	Pain assessment 7 days after surgery vs. 4 months after surgery	−3.83	<0.001	0.61
G4	Pain assessment 1 day after surgery vs. 3 days after surgery	−0.24	0.810	0.04
	Pain assessment 1 day after surgery vs. 7 days after surgery	−3.16	0.002	0.48
	Pain assessment 1 day after surgery vs. 4 months after surgery	−4.02	<0.001	0.61
	Pain assessment 3 days after surgery vs. 7 days after surgery	−3.61	<0.001	0.54
	Pain assessment 3 days after surgery vs. 4 months after surgery	−4.02	<0.001	0.61
	Pain assessment 7 days after surgery vs. 4 months after surgery	−3.94	<0.001	0.59
G5	Pain assessment 1 day after surgery vs. 3 days after surgery	−0.38	0.704	0.06
	Pain assessment 1 day after surgery vs. 7 days after surgery	−3.16	0.002	0.51
	Pain assessment 1 day after surgery vs. 4 months after surgery	−3.73	<0.001	0.62
	Pain assessment 3 days after surgery vs. 7 days after surgery	−3.39	<0.001	0.55
	Pain assessment 3 days after surgery vs. 4 months after surgery	−3.73	<0.001	0.62
	Pain assessment 7 days after surgery vs. 4 months after surgery	−3.39	<0.001	0.57

*z*—value of the test statistic; *p*—statistical significance; r—strength of effect index.

**Table 9:** Results of analysis by Student’s *t* test for one sample comparing alveolar fractal dimensions to the mean fractal dimension of the reference bone.

Group	Fractal Dimension Compared	N	M	SD	*t*	df	*p*	95% CI	
								LL	UL
G0	Apex of the alveolus	22	1.42	0.08	−1.76	21	0.093	−0.07	0.01
	Mid-alveolar region	22	1.41	0.06	−3.59	21	0.002	−0.07	−0.02
	Entrance to the alveolus	22	1.36	0.08	−5.97	21	<0.001	−0.13	−0.06
	Averaged apex and centre fractal value	22	1.42	0.06	−3.21	21	0.004	−0.07	−0.01
	Averaged fractal value of apex, middle and entrance	22	1.40	0.05	−5.90	21	<0.001	−0.08	−0.04
G1	Apex of the alveolus	18	1.47	0.06	0.77	17	0.450	−0.02	0.04
	Mid-alveolar region	18	1.44	0.07	−0.71	17	0.488	−0.04	0.02
	Entrance to the alveolus	18	1.39	0.05	−5.10	17	<0.001	−0.09	−0.04
	Averaged apex and centre fractal value	18	1.46	0.05	−0.02	17	0.985	−0.02	0.02
	Averaged fractal value of apex, middle and entrance	18	1.43	0.03	−2.77	17	0.013	−0.04	−0.01
G2	Apex of the alveolus	17	1.45	0.06	−0.52	16	0.613	−0.04	0.02
	Mid-alveolar region	17	1.44	0.06	−1.23	16	0.236	−0.05	0.01
	Entrance to the alveolus	17	1.39	0.07	−3.90	16	0.001	−0.10	−0.03
	Averaged apex and centre fractal value	17	1.44	0.05	−1.09	16	0.293	−0.04	0.01
	Averaged fractal value of apex, middle and entrance	17	1.43	0.04	−2.77	16	0.014	−0.05	−0.01
G3	Apex of the alveolus	18	1.44	0.08	−1.16	17	0.264	−0.06	0.02
	Mid-alveolar region	18	1.47	0.07	1.03	17	0.318	−0.02	0.05
	Entrance to the alveolus	18	1.38	0.06	−5.10	17	<0.001	−0.11	−0.04
	Averaged apex and centre fractal value	18	1.45	0.05	−0.16	17	0.878	−0.03	0.02
	Averaged fractal value of apex, middle and entrance	18	1.43	0.04	−2.66	17	0.016	−0.05	−0.01
G4	Apex of the alveolus	21	1.45	0.10	−0.29	20	0.775	−0.05	0.04
	Mid-alveolar region	21	1.42	0.07	−1.95	20	0.066	−0.06	0.00
	Entrance to the alveolus	21	1.38	0.07	−4.96	20	<0.001	−0.10	−0.04
	Averaged apex and centre fractal value	21	1.44	0.06	−1.44	20	0.165	−0.05	0.01
	Averaged fractal value of apex, middle and entrance	21	1.42	0.05	−3.43	20	0.003	−0.06	−0.01
G5	Apex of the alveolus	18	1.49	0.09	1.71	17	0.105	−0.01	0.08
	Mid-alveolar region	18	1.48	0.07	1.18	17	0.254	−0.02	0.06
	Entrance to the alveolus	18	1.40	0.08	−2.97	17	0.009	−0.10	−0.02
	Averaged apex and centre fractal value	18	1.48	0.07	1.72	17	0.104	−0.01	0.06
	Averaged fractal value of apex, middle and entrance	18	1.46	0.06	−0.05	17	0.963	−0.03	0.03
Total	Apex of the alveolus	114	1.45	0.08	−0.57	113	0.572	−0.02	0.01
	Mid-alveolar region	114	1.44	0.07	−2.03	113	0.045	−0.03	<−0.01
	Entrance to the alveolus	114	1.38	0.07	−11.31	113	<0.001	−0.09	−0.06
	Averaged apex and centre fractal value	114	1.45	0.06	−1.61	113	0.110	−0.02	<0.01
	Averaged fractal value of apex, middle and entrance	114	1.42	0.05	−6.62	113	<0.001	−0.04	−0.02

N—number of observations; M—mean; SD—standard deviation; *t*—value of test statistic; df—degrees of freedom; *p*—statistical significance; CI—confidence interval for the difference between means; LL and UL—lower and upper limits of the confidence interval.

**Table 10:** Results of Kruskal–Wallis H analysis testing the dependence of the degree of wound closure on the application of tissue engineering and photobiomodulation methods.

Group	Mean Rank	M	Me	SD	H(df)	*p*	η^2^
G0	64.09	3.73	4.00	0.77	4.22	0.518	<0.01
G1	57.73	3.30	4.00	1.38			
G2	62.63	3.68	4.00	0.82			
G3	53.58	3.25	4.00	1.21			
G4	62.41	3.55	4.00	1.14			
G5	68.63	3.89	4.00	0.46			

M—mean; Me—Median; SD—standard deviation; H(df)—value of test statistic and degrees of freedom; *p*—statistical significance; η^2^—effect strength index.

**Table 11:** Results of chi-square analysis of independence testing for differences in rates of complete wound closure between patients with different tissue engineering and photobiomodulation methods used.

Group	Incomplete Wound Closure		Complete Wound Closure				
	N	%	N	%	χ^2^(5)	*p*	Vc
G0	18	81.8%	4	18.2%	4.54	0.474	0.19
G1	15	75.0%	5	25.0%			
G2	16	84.2%	3	15.8%			
G3	14	70.0%	6	30.0%			
G4	18	81.8%	4	18.2%			
G5	18	94.7%	1	5.3%			
Total	99	81.1%	23	18.9%			

N—number of observations; χ^2^—chi-square test result; *p*—statistical significance; Vc—strength of effect index.

**Table 12:** Postoperative adverse events by randomised group.

Event	G0	G1	G2	G3	G4	G5
Alveolitis, *n*	2	1	1	2	1	0
Infection requiring antibiotics, *n*	0	2	2	0	0	0
Postoperative bleeding requiring intervention, *n*	0	0	0	0	0	0
Persistent neurological symptoms, *n*	0	0	0	0	0	0

## Data Availability

The original contributions presented in the study are included in the article, further inquiries can be directed to the corresponding authors.
